# Adenocarcinoma of Stump Appendicitis: An Extremely Rare Pathology – A Literature Review

**DOI:** 10.7759/cureus.9482

**Published:** 2020-07-30

**Authors:** Mohannad Al-Tarakji, Syed Muhammad Ali, Mohamed Khalaf, Fakhar Shahid, Sara Saeed I Mohamed

**Affiliations:** 1 Acute Care Surgery, Hamad Medical Corporation, Doha, QAT; 2 Acute Care Surgery, Hamad General Hospital, Doha, QAT; 3 Surgery, Weill-Cornell Medical School, Doha, QAT; 4 General Surgery, Hamad Medical Corporation, Doha, QAT; 5 Medicine, Hamad Medical Corporation, Doha, QAT

**Keywords:** stump appendicitis, adenocarcinoma, appendix

## Abstract

Emergency appendectomy for acute appendicitis is the most common emergency surgical procedure performed all over the world. While amputating the appendix from the cecum, a small (usually less than 5 mm) stump is left behind. Below this, the suture or stapler is applied to secure the base of the appendix, which is now known as a stump. Stump appendicitis, the inflammation of appendiceal remnant after an appendectomy, is a rare phenomenon. Additionally, the incidence of adenocarcinoma in the stump of the appendix is also extremely rare and can present with the symptoms of appendicitis. Adenocarcinoma in stump appendectomy patients can present from 5-43 years after the index appendectomy surgery. The majority of patients present with symptoms similar to that of appendicitis, with right lower quadrant abdominal pain, usually diagnosed via CT scans, after which they undergo stump appendectomy. The diagnosis of adenocarcinoma is only made on the histopathology report. We engaged in a review of the relevant literature published in the English language for the last 100 years. This was conducted by reviewing Google Scholar, PubMed, and MEDLINE® databases, as well as references to all related articles. There are only six cases reported in the literature, which shows the rarity of this condition. Regarding the appropriate treatment for this rare entity, multi-disciplinary team discussions should be carried out for optimum management of the individual patients. Right hemicolectomy is the recommended procedure for all patients, and prognosis depends on the staging of the disease.

## Introduction and background

Adenocarcinoma of the appendix is a rare gastrointestinal malignancy [1]. Diagnosis is usually made postoperatively, based on the histopathology report, and management is usually performed with right hemicolectomy based on staging. The incidence of adenocarcinoma post appendectomy in the remnant of the appendix (or stump) is extremely rare with a low threshold for suspicion due to the rarity of both pathologies. Very few cases have been reported in the last 100 years in the literature in English, and we reviewed all of them to highlight this rare condition.

## Review

Methods

Google Scholar, PubMed, MEDLINE®, and references to all related articles were searched with the keywords mentioning stump as well as recurrent, residual, incomplete, appendicitis, adenocarcinoma, and appendectomy. More than 210 case reports were found in the literature of which 197 were in English. These articles discussed stump appendicitis after a history of appendectomy in the past; out of these, six cases of adenocarcinoma were discussed. The data was collected, and the histopathologic diagnosis of adenocarcinoma was determined to be the inclusion criteria. The major limitation was the lack of follow-up period, as it was mentioned only in one case.

Results

Only six cases of adenocarcinoma were identified in the literature in the English language. The features of presentation, time after the first/index appendectomy, and further management of the patients are discussed in this review article. All the patients were above 60 years except for one who was 40 years old. The shortest interval after index surgery was found to range from 5-43 years. All patients underwent right hemicolectomy except for one who refused surgery. Follow-up for two years without incident was mentioned in one case.

Discussion

Appendiceal primary cancers are very rare, and studies from the USA report an incidence rate of 1.2 patients per 100,000 people in a year [1]. Acute appendicitis is the most common clinical presentation of patients harboring malignant neoplasms of the appendix. Diagnosis is usually incidental after the histopathological examination has been carried out for all appendectomy surgeries [1].

The peak age of primary malignant neoplasm of the appendix ranges from 55-65 years; however, malignant carcinoids affect a slightly younger population, usually in their third decade of life (mean age: 38 years). Carcinoid is by far the most common type of tumor of the appendix; it is followed by mucinous cystadenocarcinoma, adenocarcinoma, lymphosarcoma, paraganglioma, and granular-cell tumors, which all account for an overall incidence of 10-20% [2]. Increasing age and complicated appendicitis are the known risk factors for the appendiceal neoplasm, and some of the studies have shown an incidence as high as 29% where tumors developing in cases of complicated appendicitis treated by conservative management and interval appendectomy [3-7].

Adenocarcinoma of the appendix is a rare disease, which accounts for 0.5% of all gastrointestinal cancers, and has been found in only 0.08% in appendices removed for disease, incidentally, or at autopsy [8-11]. The first authentic case report of primary carcinoma of the appendix was reported in 1882 by Beger [12]. Acute appendicitis is the most common clinical presentation of adenocarcinoma of the appendix as luminal blockage by neoplastic growth and superimposed bacterial infection leads to classical signs and symptoms [13].

The diagnosis cannot be determined until laparotomy/laparoscopy or pathological evaluation of the appendectomy specimen is performed. The recommended treatment modality for appendiceal adenocarcinoma is oncologic right hemicolectomy as the condition is only diagnosed after histopathological examination of the specimen of the appendix [10]. An uncommon variety of appendiceal adenocarcinoma involves a post-appendectomy stump. De Ruyter reported the first case in which, at autopsy, a carcinoma was found to have developed in the stump of the appendix, which had been removed six years ago [14]. Rose et al. first described the stump appendicitis in 1945 as a long-term complication of surgical removal of the appendix [15,16]. It is defined as appendiceal tissue that remained after performing routine appendectomy as a stump, which may develop obstruction and inflammation after a varying interval following the index operation and can present clinically as acute appendicitis. A total of 197 cases have been reported in the literature in English [16-19]. Due to the rarity of the condition, it is not considered in the differential diagnosis of the right lower quadrant abdominal pain when the patient had already undergone an appendectomy; hence, the diagnosis is often delayed, which increases the incidence of perforation, need for more radical surgery, prolonged hospital stay, and postoperative morbidity [18].

Since the recognition of both pathologies of malignancy in the appendix and reoperation of symptomatic stump appendicitis are two different entities, only six cases of combined pathologies were identified in the literature in English (Table 1). All patients had an open appendectomy, and the age at presentation was in the same range as reported for this pathology. The interval between appendectomy and adenocarcinoma varied from 5-43 years. All patients, except one, were above 50 years of age and presented with either right lower quadrant pain or features of acute appendicitis [20-23]; the management offered in all cases was oncological right hemicolectomy. The second surgery may be quite difficult in the presence of adhesions from previous explorations and the presence of peritoneal metastases. The diagnosis of adenocarcinoma was based on postoperative histopathology of the specimen. A two-year follow-up of case number 1 showed no symptoms, while the rest of the case reports did not mention any follow-up.

**Table 1:** Data of all patients found in the literature in English

Serial number	Case title	Year reported	Journal	Author	Age at presentation (years)	interval period (years)	Type of malignancy	Presenting symptoms of the stump	Diagnoses of malignancy	Procedure	Outcome
1	Adenocarcinoma of the appendix: an unusual case and review [11]	1976	Diseases of the Colon & Rectum	Gamble HA 2nd	62	43	Adenocarcinoma of appendiceal stump	Right lower quadrant pain, weight loss, and decreased appetite	Postoperative	Laparotomy of a cecocecal intussusception. A right hemicolectomy performed	No complications at 2-year follow-up
2	Papillary cystadenocarcinoma of the appendiceal stump with mucocele and peritoneal metastases [20]	1989	Pathology	Yeong et al.	54	25	Papillary cystadenocarcinoma arising in an appendiceal stump and associated with peritoneal metastases	Painless abdominal mass of 5-year duration	Postoperative	Laparotomy and right hemicolectomy	Not mentioned
3	Adenocarcinoma of appendiceal stump [9]	1990	The Southern Medical Journal	Van fleet et al.	58	39	Adenocarcinoma (3 cm in stump)	Abdominal pain (vague)	Postoperative	Right hemicolectomy	uneventful
4	Adenocarcinoma of the appendiceal stump developing 23 years after an appendectomy [21]	1990	The American Journal of Gastroenterology	Kashiwagi et al.	40	23	Adenocarcinoma	Right lower quadrant abdominal pain, fever, and anorexia	Postoperative	Ileocecal resection of 5-cm, elastic, firm mass behind the ascending colon	Not mentioned
5	Appendiceal mucocele due to mucinous cystadenocarcinoma arising from the appendiceal stump: preoperative diagnosis based on the ‘‘onion skin sign’’ [22]	2012	Japanese Journal of Radiology	Ozgür et al.	71	10	Mucinous adenocarcinoma	Abdominal pain, nausea, and vomiting	Postoperative	Exploratory laparotomy and right hemicolectomy	Not mentioned
6	A rare case of appendiceal stump adenocarcinoma and review of literature [23]	2014	Kuwait Medical Journal	Xiong et al.	72	5	Adenocarcinoma	A 3-month history of repeated constipation and vomiting	Postoperative	Exploratory laparotomy and stump appendectomy with 2.5-3 cm diameter of stump mass	Patient refused right hemicolectomy, discharged home

Limitations

We could include only a few numbers of cases, as the condition is very uncommon, and only English language reports were considered. Moreover, the follow-up period was mentioned in only one case.

## Conclusions

Stump appendicitis is a rare presentation after an appendectomy, and it might be related to inflammation or malignancy. Adenocarcinoma of the stump, despite its rarity, must be considered in the differential diagnosis for patients with post-appendectomy right lower abdominal pain, especially in those who are of older age. Diagnosis is usually made by CT abdomen, and surgery should be offered in all cases of appendicitis as part of the management for diagnosis or treatment and for decreasing the risk of malignancy. A consultation involving a multi-disciplinary team should be considered for the optimum management of patients, which usually involves procedures like CT abdomen and chest with tumor markers, followed by right hemicolectomy whenever there is adenocarcinoma in the stump of an appendix.

## References

[1] Kelly KJ (2015). Management of appendix cancer. Clin Colon Rectal Surg.

[2] Ruoff C, Hanna L, Zhi W, Shahzad G, Gotlieb V, Saif MW (2011). Cancers of the appendix: review of the literatures. ISRN Oncol.

[3] Loftus TJ, Raymond SL, Sarosi GA Jr (2017). Predicting appendiceal tumors among patients with appendicitis. J Trauma Acute Care Surg.

[4] Wright GP, Mater ME, Carroll JT, Choy JS, Chung MH (2015). Is there truly an oncologic indication for interval appendectomy?. Am J Surg.

[5] Furman MJ, Cahan M, Cohen P, Lambert LA (2013). Increased risk of mucinous neoplasm of the appendix in adults undergoing interval appendectomy. JAMA Surg.

[6] Carpenter SG, Chapital AB, Merritt MV, Johnson DJ (2012). Increased risk of neoplasm in appendicitis treated with interval appendectomy: single-institution experience and literature review. Am Surg.

[7] Lietzén E, Grönroos JM, Mecklin JP (2019). Appendiceal neoplasm risk associated with complicated acute appendicitis-a population based study. Int J Colorectal Dis.

[8] Nilecki SS, Wolff BG, Schlinkert R, Sarr MG (1994). The natural history of surgically treated primary adenocarcinoma of the appendix. Ann Surg.

[9] Van Fleet RH, Shabot JM, Halpert RD (1990). Adenocarcinoma of the appendiceal stump. South Med J.

[10] Douglas SS, David IS (2006). Appendix and appendectomy. Maingot’s Abdominal Operations, 11th Edition.

[11] Gamble HA 2nd (1976). Adenocarcinoma of the appendix: an unusual case and review. Dis Colon Rectum.

[12] Beger A (1882). A case of cancer of the appendix. (Article in German). Berl Klin Wochenschrift.

[13] Hartley JE, Drew PJ, Qureshi A, MacDonald A, Monson JR (1996). Primary adenocarcinoma of the appendix. J R Soc Med.

[14] de Ruyter G (1903). About carcinoma development. (Article in German). Arch Klin Chir (Berl).

[15] Liang MK, Lo HG, Marks JL (2006). Stump appendicitis: a comprehensive review of literature. Am Surg.

[16] Kanona H, Al Samaraee A, Nice C, Bhattacharya V (2012). Stump appendicitis: a review. Int J Surg.

[17] Leff DR, Sait MR, Hanief M, Salakianathan S, Darzi AW, Vashisht R (2012). Inflammation of the residual appendix stump: a systematic review. Colorectal Dis.

[18] Subramanian A, Liang MK (2012). A 60-year literature review of stump appendicitis: the need for a critical view. Am J Surg.

[19] Papi S, Pecchini F, Gelmini R (2014). Stump appendicitis: a rare and unusual complication after appendectomy. Case report and review of the literature (Epub ahead of print). Ann Ital Chir.

[20] Yeong ML, Clark SP, Stubbs RS (1989). Papillary cystadenocarcinoma of the appendiceal stump with mucocele and peritoneal metastases. Pathology.

[21] Kashiwagi H, Kawamitsu M, Shikano S, Katayanagi T, Shouji M (1990). Adenocarcinoma of the appendiceal stump developing 23 years after an appendectomy. Am J Gastroenterol.

[22] Ozgür A, Cabuk G, Nass Duce M, Cereb Tombak M, Esen K (2012). Appendiceal mucocele due to mucinous cystadenocarcinoma arising from the appendiceal stump: preoperative diagnosis based on the “onion skin sign”. Jpn J Radiol.

[23] Xiong L, Li TG, Zhao H (2014). A rare case of appendiceal stump adenocarcinoma and review of literature. Kuwait Med J.

